# A Novel Method to Synthesize Co/Fe_3_O_4_ Nanocomposites with Optimal Magnetic and Microwave Performance

**DOI:** 10.3390/nano12162764

**Published:** 2022-08-12

**Authors:** Chi Zhang, Yao Wang, Yun Chen, Yatao Wang, Peng Wang, Qiong Wu

**Affiliations:** 1 Faculty of Materials and Manufacturing, Beijing University of Technology, Key Lab of Advanced Functional Materials, Ministry of Education, Beijing 100124, China; 2 Industry Development and Promotion Center, Ministry of Industry and Information Technology of China, Beijing 100846, China; 3 Department of Physics, School of Physics and Materials Science, Anhui University, Hefei 230601, China

**Keywords:** magnetic materials, nanoparticles, nanocomposites, electromagnetic

## Abstract

The magnetic interactions between neighboring magnetic nanoparticles make the synthesis of nanocomposites made of two kinds of magnetic nanoparticles extremely difficult. In this paper, to achieve an effective nanocomposite of Co and Fe_3_O_4_ nanoparticles, a special urchin-like Co nanomatrix was used to prepare the Co/Fe_3_O_4_ nanocomposites. The Fe_3_O_4_ nanoparticles are evenly embedded into the branches of the C_O_ clusters, bringing the two types of particles into close contact and ensuring the optimal magnetic and microwave properties. The electromagnetic (EM) parameters at 1–18 GHz and the magnetic loss tangents can be effectively modulated, and the absorption frequency bands of the EM waves are shifted to the X-Ku bands (8–18 GHz) from the S-C bands (2–8 GHz) after the Fe_3_O_4_ nanoparticles are compounded.

## 1. Introduction

**Figure 1:**
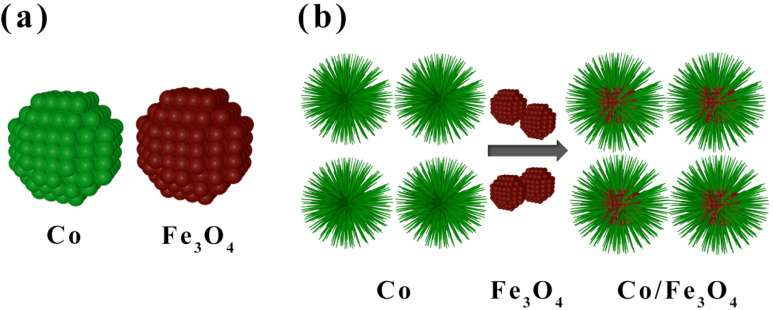
(**a**) Distribution morphology of Co and Fe_3_O_4_ magnetic nanoparticles. (**b**) A schematic diagram of the synthesis of Co/Fe_3_O_4_ nanocomposites.

Electromagnetic (EM) materials have a broad range of applications in electronics and the military (e.g., aircraft stealth). In recent years, concern about the harm of EM waves to human health has led to increasing attention [1,2,3,4,5,6,7,8]. The high performance of microwave-absorbing materials (MAMs) includes not only their strong absorption, small size, and wide operating frequency range, but also their excellent mechanical properties, high chemical stability, low temperature coefficient, and low cost [9,10,11,12,13]. To obtain MAMs with comprehensive performance, the nanocomposites of different materials such as magnetic metals and ferrite, functional ceramics, conductive polymers, and carbon materials are particularly promising options [14,15,16,17,18,19,20,21,22,23]. For example, the nanocomposites of graphene and magnetic materials feature the advantages of excellent microwave absorption ability and low density, but the high price of graphene limits its practical applicability [24,25,26,27,28,29,30,31,32]. Magnetic metal (Fe, Co, Ni) and ferrite (NiFe_2_O_4_, CoFe_2_O_4_, NiZnFe_2_O_4_, MnZnFe_2_O_4_, Fe_3_O_4_, etc.) nanocomposites, such as Co/Fe_3_O_4_, are cheaper compared with graphene when used as EM absorption materials on a large scale. As an EM function material, the dispersity of particles usually has an important effect on the frequency spectrums of the EM parameters [33,34,35,36,37]. A good particle dispersity in nanocomposites will contribute to the improvement of the high frequency properties and the EM absorption performance [38,39,40]. However, the spontaneous magnetic interactions between neighboring magnetic nanoparticles will lead to particle agglomeration, which will make the nanocomposite synthesis for two kinds of magnetic nanoparticles extremely difficult [41,42,43,44,45]. The ideal distribution of nanocomposites is difficult to achieve, as illustrated in Figure 1a, even if the sizes of the two kinds of magnetic nanoparticles are well controlled. Therefore, a study of the Co/Fe_3_O_4_ nanocomposites with good dispersity is necessary.

In this work, to achieve an effective nanocomposite made of two kinds of magnetic nanoparticles, a special urchin-like Co nanomatrix was used to prepare the Co/Fe_3_O_4_ nanocomposites. The Fe_3_O_4_ nanoparticles were evenly embedded in the branches of Co urchin particles, which led to intimate contact between the two types of particles, so that the optimal magnetic and microwave performances were ensured. The schematic diagram of the synthesis of the Co/Fe_3_O_4_ nanocomposites is illustrated in Figure 1b. For the nanocomposite of Co and Fe_3_O_4_, an adjustable EM parameter, magnetic loss angle tangents, and wave absorption performance were achieved.

## 2. Materials and Methods

### 2.1. Synthesis of Co/Fe_3_O_4_ Composite Particles

The Co clusters were first synthesized using a solvothermal chemical process. We dissolved 2.0589 g (4.5 mmol) of cobalt laurate, 0.5810 g (2.4 mmol) of hexadecylamine, and RuCl_3_ in 60 mL of 1,2-butanediol (BEG), and then added them to a Teflon container (100 mL) in a glove box with argon. Afterwards, the container was heated in an ultrasonic bath at 353 K for one hour. The Teflon container was then heated to 523 K at a rate of 10 K min^−1^ and kept at 523 K for 80 min in an autoclave reactor. After cooling to room temperature, we washed the powders with toluene and then dried them under vacuum. The Co clusters were then mixed with Fe_3_O_4_ nanoparticles at a mass ratio of 1:1, poured into anhydrous ethanol and placed in an ultrasonic bath for ultrasonic mixing for 30 min. After this, anhydrous ethanol was removed by centrifuge and the powders were drained in a vacuum drying oven to obtain the Co/Fe_3_O_4_ composite particles.

### 2.2. Characterization

X-ray diffraction (XRD, RIGAKU Ultima IV, Tokyo, Japan) measurements with a Cu-Ka wavelength X-ray source were used to identify the crystalline structure of the precursor and the final product. The microstructure and morphology were studied via scanning electron microscopy (SEM, ZEISS-SUPRA55, Oberkochen, Germany) and transmission electron microscopy (TEM, Tecnai-F20, Hillsboro, OR, USA). The magnetic performances of the nanoparticles were measured at different temperatures (300–400 K) using a vibrating sample magnetometer (VSM, Quantum Design, San Diego, CA, USA) under a maximum applied field of 30 kOe. The EM parameters of 2–18 GHz were measured using the vector network analyzer (VNA, NYSE:A, Palo Alto, CA, USA) with a coaxial method.

## 3. Results and Discussion

**Figure 2:**
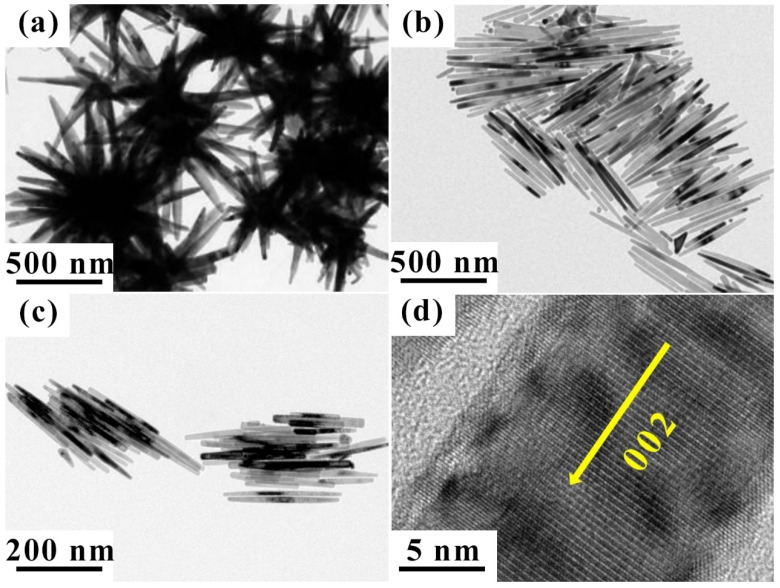
TEM images of Co nanoclusters with different n_Ru_/n_Co_ molar ratios of (**a**) 0.0, (**b**) 0.2%, and (**c**) 0.4%, and (**d**) a high-resolution TEM image of a single Co nanorod.

Figure 2 displays the morphology of the pure Co clusters with different lengths and shapes obtained from the TEM observations. RuCl_3_ is crucial in the growth of nano-crystals, as the length of the Co nano-crystals is regulated by changing the Ru/Co molar ratio. During the formation of cobalt nanorods, the effect of the trace Ru is to produce a tiny metal seed for each individual nanorod. Therefore, when the total quantity of the material is fixed, the length of each nanorod depends largely on the concentration of the seed in the medium. When the Ru addition equals 0, the urchin-like Co cluster, which is composed of many Co nanorods, is obtained, as demonstrated in Figure 2a. When the Ru/Co molar ratio is 0.2%, Figure 2b shows that the Co nanorods possess an average length of 355 nm, and are cylindrical with ellipsoid tips. When the Ru/Co molar ratio is further increased to 0.4%, the average length of the nanorods decreases to 297 nm, with almost the same diameter of 18 ± 5 nm, as demonstrated in Figure 2c. The high-resolution TEM results in Figure 2d reveal that each Co nanorod is a single crystal with a hexagonal close-packed (hcp) structure. Additionally, the long axis direction of the rod is the c-axis (002), as well as the easy magnetization direction. These kinds of single-crystal Co nanorods have strong magnetic anisotropy. The urchin-like Co cluster is well designed as a matrix to be compounded with nanoparticles.

**Figure 3:**
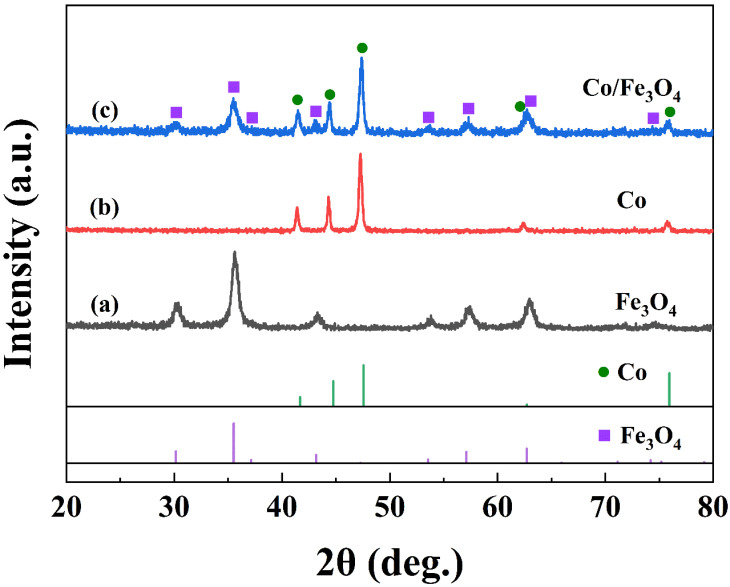
The XRD patterns of Fe_3_O_4_ nanoparticles (**a**), Co clusters (**b**), and Co/Fe_3_O_4_ composite particles (**c**) with the standard diffraction patterns of hcp-Co and Fe_3_O_4_ (JCPDS No. 05-0727, and 88-0315).

The crystalline structure of the samples obtained at different stages of the preparation process were characterized via XRD. Figure 3 shows the XRD patterns of Fe_3_O_4_ nanoparticles (Figure 3a), Co clusters (Figure 3b), and Co/Fe_3_O_4_ composite particles (Figure 3c), respectively. A typical pattern that matches with the standard pattern for hcp-Co is found in Figure 3b,c. Obviously, Fe_3_O_4_ nanoparticles and Co clusters are well preserved in the Co/Fe_3_O_4_ composite particles. Note that the broad peaks also suggest the nanostructure of the Fe_3_O_4_ and Co.

**Figure 4:**
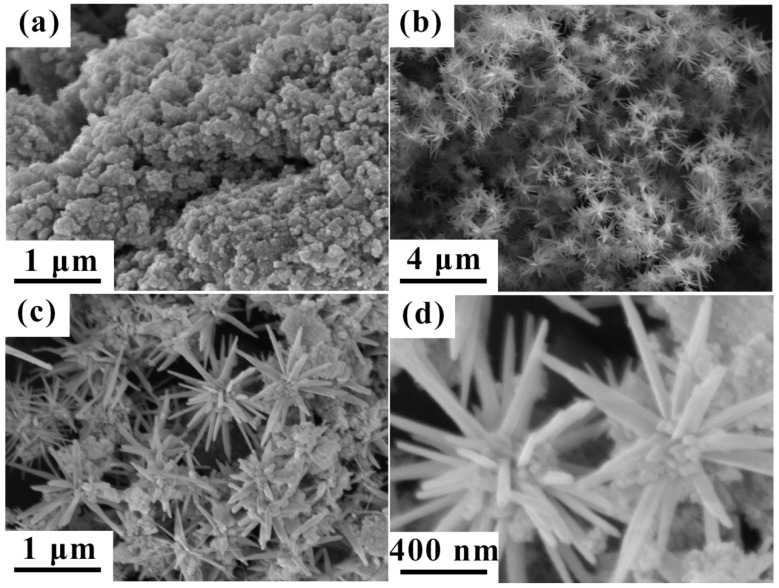
SEM images of Fe_3_O_4_ nanoparticles (**a**), Co clusters (**b**), and Co/Fe_3_O_4_ composite particles (**c**,**d**).

The microstructures of the Co and Fe_3_O_4_ composite particles were directly observed using SEM, as shown in Figure 4. Figure 4a shows the microscopic morphology of Fe_3_O_4_ particles, and the average particle size for Fe_3_O_4_ is 100 nm. Figure 4b demonstrates the morphology of the Co clusters prepared using the solvothermal chemical process. The uniform urchin-shaped Co clusters were obtained with a good distribution. This special kind of urchin-shaped Co cluster was constructed with the pre-nucleated Co seeds and long Co nanorods synthesized through axial growth from the nucleation. Figure 4c,d shows the morphology of the Co/Fe_3_O_4_ composite particles. It is obvious that the Fe_3_O_4_ particles are evenly distributed in the branches of the urchin-shaped Co clusters, indicating that the two phases were well composited.

**Figure 5:**
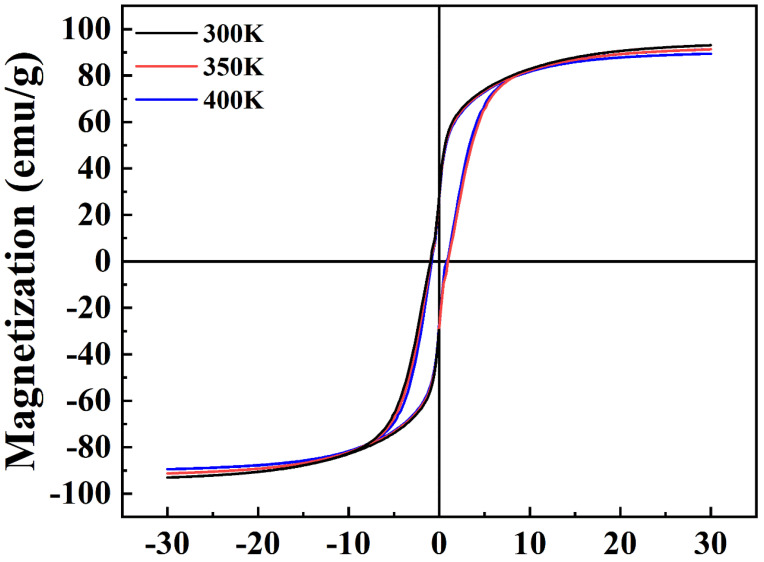
Magnetization hysteresis loops of Co/Fe_3_O_4_ composite particles at different temperatures (300, 350, and 400 K).

The magnetic performance of the Co/Fe_3_O_4_ composite particles were measured by VSM at a maximum applied magnetic field of 30 kOe, as shown in Figure 5. Figure 5 presents the hysteresis loops of composite particles at different temperatures (300, 350, and 400 K). They exhibit ferromagnetic behavior at different temperatures, with saturation magnetization (Ms) rates of 93.1 emu/g (300 K), 91.4 emu/g (350 K), and 89.3 emu/g (400 K), which are smaller than the reported Co microflakes (160 emu/g) [46], Co/CoO nanorods (143.2 emu/g) [47], and Co spheres (123 emu/g) [48]; equal or greater than the reported Fe_3_O_4_ nanoparticles (58–80 emu/g) [49,50]; and larger than the reported Co/Fe_3_O_4_ nanocomposites (63.1–72.4 emu/g) [1], ensuring a relatively high Ms and a relatively large permeability for our sample. The remanence (Mr) rates are 28.5 emu/g (300 K), 28.2 emu/g (350 K), and 27.1 emu/g (400 K), and the coercivity (Hci) rates are 0.95 kOe (300 K), 0.94 kOe (350 K), and 0.84 kOe (400 K). The magnetic properties of the composite particles change little at different temperatures, indicating that the temperature stability is good. Therefore, the Co/Fe_3_O_4_ composite is practicable over a relatively wide temperature range of 300–400 K as an EM absorption material.

**Figure 6:**
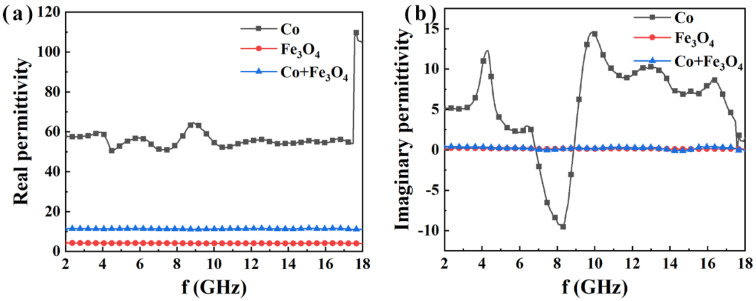
(**a**) Real (*ε*′) and (**b**) imaginary (*ε*″) parts of the permittivity curves for Co, Fe_3_O_4_, and Co/Fe_3_O_4_.

**Figure 7:**
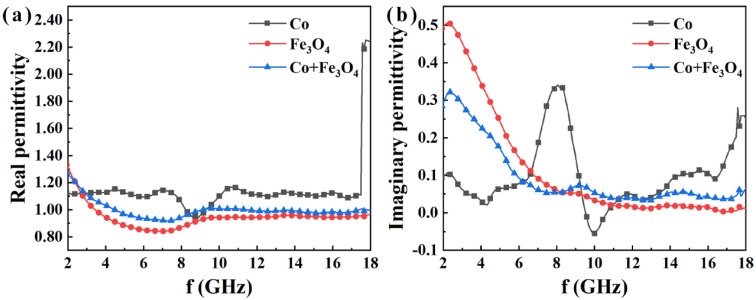
(**a**) Real (*μ*′) and (**b**) imaginary (*μ*″) parts of the Co, Fe_3_O_4_ and Co/Fe_3_O_4_ as functions of the frequency.

The electromagnetic parameters of the magnetic particles and paraffin composites were measured at 2–18 GHz with the VNA using coaxial method, as shown in Figure 6 and Figure 7, respectively. The real *ε*′ part of the complex permittivity curve (Figure 6a) displays that the *ε*′ values of the three composite samples with the same mass filling in paraffin are ranked in the following order: *ε*′ (Co) > *ε*′ (Co + Fe_3_O_4_) > *ε*′ (Fe_3_O_4_). The values of *ε*′ (Co + Fe_3_O_4_) equal approximately 10.0 across the whole testing frequency band, exactly in the range of impedance matching, which shows that the *ε*′ value of the metal Co can be effectively tailored through the Fe_3_O_4_ of the low *ε*′. The imaginary part *ε*″ of the complex permittivity (Figure 6b) reveals that the *ε*″ values of the metal Co/paraffin sample present a novel resonance behavior with the increasing frequency, which is ascribed to the unusual morphology and structure of the Co particles, leading to multiple electronic relaxation processes on the surfaces of the Co particles. After mixing with the Fe_3_O_4_ nanoparticles of high electrical resistivity, the electronic relaxation on the surface is suppressed, resulting in the low *ε*″ values in the Co/Fe_3_O_4_/paraffin sample. From the perspective of microwave absorption, on the one hand *ε*′ (Co + Fe_3_O_4_) ≈ 10.0, which is beneficial to achieving a relatively good impedance matching and EM wave absorption peak. On the other hand, the low *ε*″ (Co + Fe_3_O_4_) ≈ 0, which is adverse to enhancing the microwave attenuation (Figure 6b).

**Figure 8:**
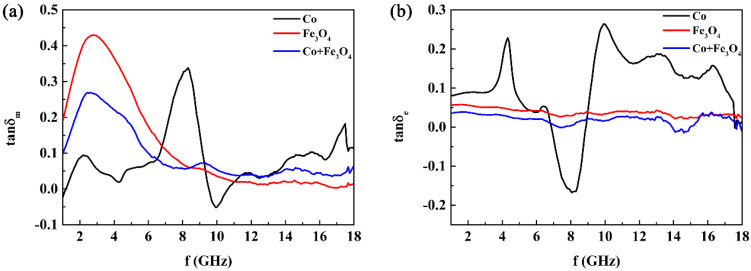
(**a**) Magnetic loss angle tangent $\left(\tan\delta_{m}\right)$ and (**b**) dielectric loss angle tangent $\left(\tan\delta_{e}\right)$.

From the real part (*μ*′) of the complex permeability (Figure 7a), it can be observed that the *μ*′ values of the three composite samples are not high compared with the usual soft magnetic alloys. This is interpreted as being due to the small particle size and low mass filling in the paraffin. Although the Co particles have a size distribution of ~2 μm (Figure 4b), the branches of Co clusters are only up to the nanoscale. In the magnetic nanoparticles, the domain structure is very minute, discontinuous, and randomly oriented, leading to low *μ*′ values. Comparatively, the metal Co/paraffin sample has a higher *μ*′ than the Co/Fe_3_O_4_/paraffin and Fe_3_O_4_/paraffin samples because of the higher magnetization intensity. Moreover, the *μ*′ value of the Co/paraffin sample is approximately 1.1 across the whole testing frequency band, indicating that the Co clusters with a branched structure should have a relatively good impedance matching degree. However, the attenuation frequency band will shift to a low-frequency region after being compounded with the Fe_3_O_4_ nanoparticles, according to the imaginary part (*μ*″) of the complex permeability curve (Figure 7b). It can be clearly seen that the *μ*″ values of Fe_3_O_4_/Co/paraffin and Fe_3_O_4_/paraffin samples are higher in the frequency range of 2–6 GHz than for the Co/paraffin sample, which demonstrates that the Fe_3_O_4_ nanoparticles are appropriate for low-frequency microwave absorption and means that the absorption frequency band can be tailored by controlling the ratio of Co to Fe_3_O_4_. Accordingly, the magnetic loss angle tangent ($\tan\delta_{m}=\mu^{\prime \prime }/\mu^{\prime }$) values of Co clusters can also be adjusted in different frequency bands using the Fe_3_O_4_ nanoparticles (Figure 8a). Three $\tan\delta_{m}$ peaks can be observed at ~2 GHz, ~8 GHz, and ~17.5 GHz in the Co/paraffin sample, which correspond to the natural resonance and the two exchange resonances. After being compounded with Fe_3_O_4_ nanoparticles, the two exchange resonance peaks disappear, since the exchange interaction between the Co nanoparticles is intercepted by the Fe_3_O_4_ nanoparticles. However, the natural resonance peak at ~2.5 GHz is enhanced, since the natural resonance frequency of Fe_3_O_4_ is located at ~3.0 GHz. Thus, the regulation and control of the Fe_3_O_4_ nanoparticles via the microwave magnetism of Co clusters involve two sides. On the one hand, the natural resonance peak will be enhanced, while the exchange resonance peaks will be weakened. Therefore, the ratio of Fe_3_O_4_ to Co clusters can be further designed according to the application frequency band. 

**Figure 9:**
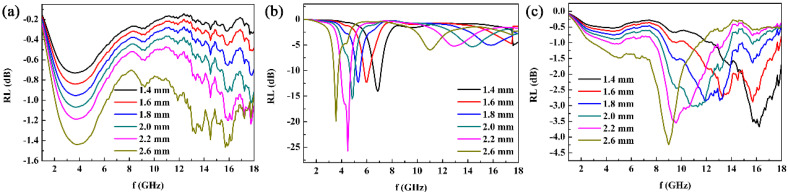
(**a**) The RL-f curves of the Fe_3_O_4_/paraffin sample, (**b**) RL-f curves of the Co/paraffin sample, and (**c**) RL-f curves of the Co/Fe_3_O_4_/paraffin sample.

The EM wave absorption performance at normal incidence over 2–18 GHz is evaluated by simulating the reflection loss (RL)-frequency (f) curves at different thicknesses using the transmission line formulas, based on the measured EM parameters, as shown in Figure 9. It can be observed that the |RL| values of the Fe_3_O_4_/paraffin sample are very low due to the weak dielectric loss ability of the Fe_3_O_4_ nanoparticles (Figure 8b). The |RL| values of the Co clusters with paraffin are relatively high. All of the RL peaks exceed −10 Db and the minimum RL value ($RL_{min}$) is up to −25.73 Db at 4.485 GHz when the absorber thickness is 2.2 mm, showing that the Co clusters are suitable for EM absorption in low-frequency bands, which can only be attributed to relatively good impedance matching, since the magnetic loss and dielectric loss are not the highest (Figure 8). If the EM parameters of the Co clusters can be effectively tailored by the Fe_3_O_4_ nanoparticles, a better and more tunable EM wave absorption performance can be expected. Figure 9c shows that the absorption peak and frequency band have been modulated to a higher frequency range, although the *RL* peaks are not strong, after the Co clusters are compounded with the Fe_3_O_4_ nanoparticles, which shows that the EM absorption performance of the Co clusters is adjustable through the Fe_3_O_4_ nanoparticles. Only the ratio of Co to Fe_3_O_4_ needs to be further designed, since the Fe_3_O_4_ is superfluous relative to the Co clusters, leading to an immoderate modulation for the ε′, a relatively low ε′ value, a dissatisfactory impedance matching degree, and *RL* peaks. We believe that strong *RL* peaks and a wide absorption frequency band will be finally achieved if the mass ratio of Co to Fe_3_O_4_ is further debugged.

## 4. Conclusions

Co clusters with specific nanostructures were synthesized using a novel technical route. In the aggregated Co nanostructures, the Fe_3_O_4_ nanoparticles were uniformly compounded, forming nanocomposite particles with a good dispersion. The crystal structure, morphology, static magnetic properties, and microwave properties were characterized or measured via XRD, SEM, VSM, and VNA, respectively. The EM parameters and magnetic loss angle tangents can be effectively modulated using the Fe_3_O_4_ nanoparticles, resulting in a tunable EM absorption peak and absorption band from the S-C bands to X-Ku bands, although they are weak, which greatly expands the application scenarios in various frequency bands, such as the mobile phone (1.8–2.7 GHz), wireless router (2.4 GHz + 5 GHz), and communication satellite (8–18 GHz) bands, when the Co/Fe_3_O_4_ nanocomposites are used as the EM absorption coatings.

## Data Availability

The data presented in this study are available on request from the corresponding author.
